# Inhibition of TLR8- and TLR4-induced Type I IFN induction by alcohol is different from its effects on inflammatory cytokine production in monocytes

**DOI:** 10.1186/1471-2172-12-55

**Published:** 2011-09-30

**Authors:** Maoyin Pang, Shashi Bala, Karen Kodys, Donna Catalano, Gyongyi Szabo

**Affiliations:** 1 Department of Medicine, University of Massachusetts Medical School, 364 Plantation Street, Worcester, MA 01605, USA; 2 Department of Medicine, Brown University School of Medicine, Rhode Island Hospital, Providence, Rhode Island 02903, USA

**Keywords:** Acute, chronic, alcohol, IFNβ, IL-10, TNF alpha

## Abstract

**Background:**

Prolonged alcohol consumption is a significant co-factor in the progression of chronic viral infections including hepatitis C and HIV, which are both single-stranded RNA viruses. Toll like receptor 8 (TLR8), a pattern recognition receptor expressed in monocytes, senses viral single stranded RNA as a danger signal and leads to the induction of Type I interferon (IFN) as well as the pro-inflammatory cytokine, tumor necrosis factor alpha (TNF alpha). Lipopolysaccharide (LPS), a Toll like receptor 4 (TLR4) ligand, was shown to affect inflammatory cell activation after alcohol consumption and in HIV and HCV infections. Here we hypothesized that alcohol exposure modulates TLR8- and TLR4-ligand-induced monocyte activation and affects both type I IFN and inflammatory cytokine induction.

**Results:**

The TLR8 ligand, CL075, as well as the TLR4 ligand, LPS, resulted in a significant induction of TNF alpha both at the mRNA and protein levels in human monocytes. We found that both acute and prolonged alcohol treatment resulted in inhibition of type I IFN induction by either TLR8 or TLR4 ligands in human monocytes at the protein and mRNA levels. In contrast to Type I IFN production, the effects of acute and prolonged alcohol were different on inflammatory cytokine activation after TLR8 or TLR4 ligand stimulation. Acute alcohol inhibited TLR8- or TLR4-induced TNF alpha protein and mRNA induction while it augmented IL-10 production in monocytes. In contrast, prolonged alcohol treatment augmented TNF alpha without affecting IL-10 production significantly in response to either TLR8 or TLR4 ligand stimulation.

**Conclusions:**

These novel results suggest first, that alcohol has a profound inhibitory effect on Type I IFN induction regardless of intracellular (TLR8) or cell surface-derived (TLR4) danger signals. Second, both acute and prolonged alcohol exposure can inhibit antiviral Type I IFN pathway activation. Third, the opposite effects of acute (inhibitory) and prolonged alcohol (augmentation) treatment on pro-inflammatory cytokine activation extend to TLR8-induced signals beyond the previously shown TLR4/LPS pathway.

## Background

The immunoregulatory effects of acute and chronic alcohol use have been linked to negative clinical outcomes including prolonged recovery after trauma or burn injury, elective surgery, liver disease and infections [1-4]. Excessive alcohol consumption was also identified as an independent risk factor in hepatitis C virus (HCV) infection that leads to chronic infection in up to 80% of cases [5,6]. Excessive alcohol use also affects outcomes in HIV infection and predisposes to advanced disease [7,8]. Both HCV and HIV are single-stranded RNA viruses that induce anti-viral innate immune responses via host pathways that recognize the viral "danger signals" [9-11]. Toll-like receptor 8 (TLR8) can sense single-stranded RNA from both HIV and HCV viruses and triggers downstream signaling to induce production of Type I IFNs and pro-inflammatory cytokines [12-14]. TLR8 is expressed in monocytes and macrophages and it is localized intracellularly in the endosomes where it is positioned to be expose to viral danger signals [15].

Limited numbers of previous studies evaluated the effects of alcohol on virus-induced immune responses and mostly showed decreased antiviral immune responses [4,16-18]. In hepatoma cells, alcohol was shown to affect innate antiviral pathways and increase HCV replication in human liver cells [19]. The effects of alcohol exposure on anti-viral immune responses are yet to be delineated in human immune cells that contribute to the production of both Type I IFNs and inflammatory cytokines [9].

In addition to viral danger signals, gut-derived lipopolysaccharide (LPS), a ligand of Toll like receptor 4 (TLR4), has been shown to play a role in HIV and HCV infection as well as in complications of chronic alcohol exposure [20-22]. It has been shown that serum/plasma LPS levels are elevated in patients with untreated HIV infection and a moderate increase in serum LPS was also reported in patients with chronic HCV infection [23,24]. It has been postulated that the source of the circulating LPS is the gut due to increased gut permeability [23]. Increased gut permeability and an increase in circulating LPS levels are the central components in the pathogenesis of alcoholic hepatitis [25,26]. Thus, LPS/TLR4 stimulation has direct clinical relevance to HIV and HCV as well as alcohol consumption.

The effects of alcohol on LPS/TLR4 signaling were evaluated in numerous previous studies, however, the effects of alcohol on TLR4-induced IFN responses are yet to be defined. Here, we tested the hypothesis that acute and prolonged alcohol exposures modulate pro-inflammatory cytokines as well as Type I IFN induction in response to TLR8 or TLR4 ligand stimulation.

## Results

### TLR8- and TLR4-induced Type I IFN production is inhibited by acute and prolonged alcohol treatment in human monocytes

**Figure 1:**
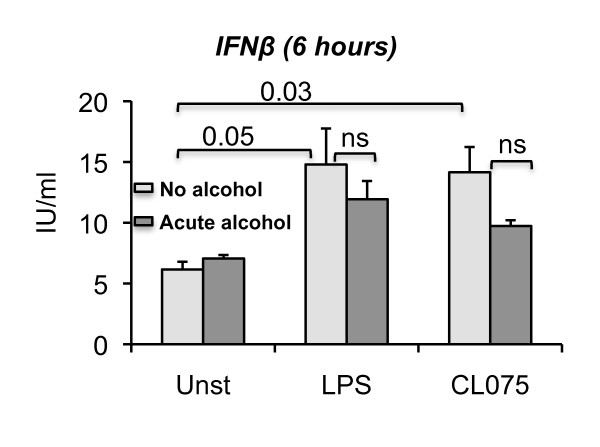
**Decreased IFNβ levels in monocytes stimulated with acute alcohol and TLR8 or TLR4 ligand**. Human monocytes (n = 3) were isolated as described in the methods. Cells were either treated or not with 25 mM ethanol or 2.5 ug/ml CL075 (TLR8 ligand) or 0.1 ug/ml LPS (TLR4 ligand) for 6 hours. Some cells were stimulated with a combination of 25 mM ethanol and 2.5 ug/ml CL075 or 25 mM ethanol and 0.1 ug/ml LPS. The cell-free supernatants were collected and IFNβ production was measured by ELISA. Error bars represent ± SEM. unst: unstimulated, ns: non significant.

Innate immune responses by circulating monocytes and tissue macrophages are triggered by viral and bacterial danger signals to induce Type I IFNs and inflammatory cascade activation [4,17]. TLR8, expressed in the endosome senses single-stranded RNA while TLR4, expressed on the cell membrane is activated by bacterial lipopolysaccharide [27]. Here we tested the hypothesis that alcohol interferes with Type I IFN production in response to viral or bacterial pathogen-derived signals. Human peripheral blood monocytes were stimulated with a TLR8 or TLR4 ligand in the presence or absence of 25 mM alcohol. This in vitro treatment represents acute alcohol exposure in humans as 25 mM alcohol approximates 0.1 g/dl blood alcohol level often found after consumption of 4-5 drinks in non-alcoholic individuals [28]. Monocyte stimulation with CL075 (TLR8 ligand) or LPS (TLR4 ligand) resulted in a significant increase in IFNβ production at 6 hours after stimulation while alcohol treatment alone did not affect baseline IFNβ production (Figure 1). Acute alcohol treatment during the TLR8 and TLR4 stimulation resulted in a decreasing trend in IFNβ production in monocytes but this did not reach statistical significance (Figure 1).

Considering that chronic alcohol consumption in humans often involves sustained alcohol exposure, we next evaluated the effects of prolonged alcohol treatment on IFNβ production. Monocytes were exposed to 25 mM alcohol for 7 days followed by TLR8 and TLR4 ligand stimulation. The alcohol concentration in the supernatant was measured using the Analox Alcohol Analyzer (Analox Instruments) and ranged from 23 mM to 21 mM over the 7-day period (Additional file 1, Table S1). This result is in accordance with our previous study where we reported that alcohol concentrations vary between 24 to 20 mM [28]. To rule out the possibility that prolonged alcohol treatment has any adverse effect on cell viability, we performed an MTT assay (Promega) and found no difference in the viability of alcohol -naïve and -treated cells as shown in Additional file 2, Figure S1. Recently, it has been shown that alcohol (25 mM) does not influence the human peripheral mononuclear cells viability when either incubated for 24 hours or 48 hours and our results support this finding [29].

**Figure 2:**
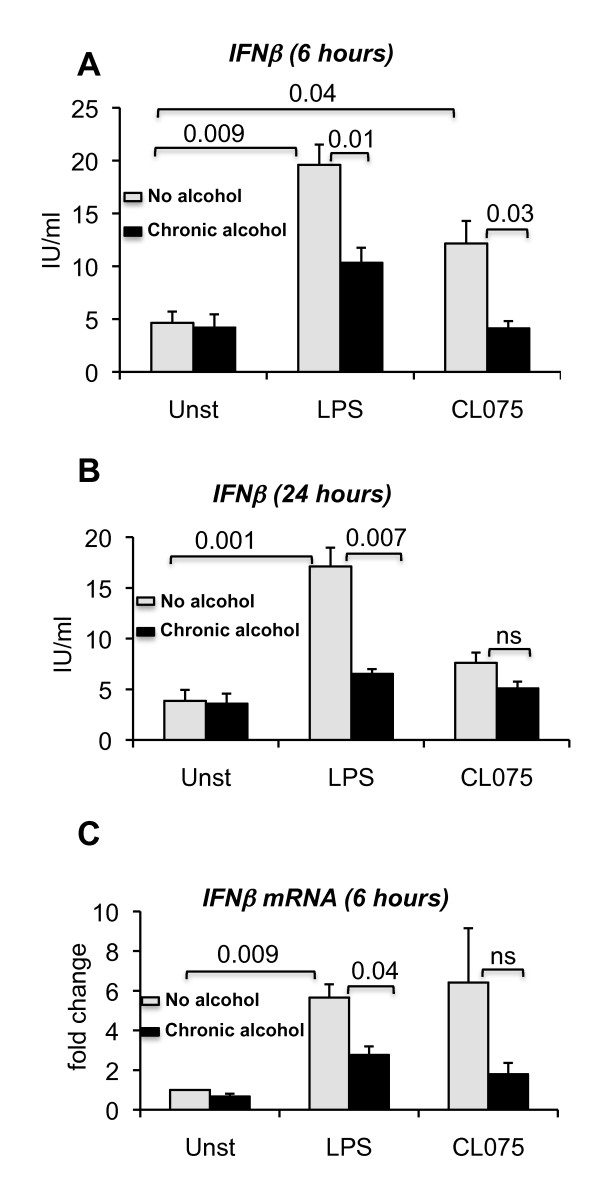
**Prolonged alcohol exposure down regulates TLR8- or TLR4- induced IFNβ expression both at protein and mRNA levels**. Human monocytes (n = 5 or 6) were either treated or not with 25 mM ethanol for 7 days, 2.5 ug/ml CL075 or 0.1 ug/ml LPS for 6 or 24 hours and alcohol treated cells were further stimulated or not with CL075 or LPS for 6 hours (A and C) or 24 hours (B). The cell-free supernatants were collected and analyzed for IFNβ production by ELISA (A and B, n = 6). Total RNA was extracted from the cells and real time PCR was performed to quantify the IFNβ gene expression (C, n = 5). Error bars represent ± SEM. unst: unstimulated, ns: non significant.

Here we found that prolonged alcohol treatment alone did not have any affect on baseline IFNβ production (Figure 2A and 2B). However, we found significant attenuation of both TLR8- and TLR4-induced IFNβ production in prolonged alcohol-treated monocytes at 6 hours (Figure 2A). Inhibition of IFNβ production was sustained in TLR4-stimulated cells even after 24 hours (Figure 2B). These data suggested that in the presence of prolonged alcohol treatment, IFNβ production is decreased in monocytes whether induced by TLR8 or TLR4 stimulation. To further assess the mechanisms of alcohol-induced inhibition of IFNβ production, we evaluated the mRNA levels of IFNβ and found that both TLR8 and TLR4-induced IFNβ mRNA levels were decreased in monocytes after prolonged alcohol treatment (Figure 2C). These data suggested that prolonged alcohol treatment inhibits type I IFN induction via TLR8 or TLR4 mediated pathways and affects IFNβ mRNA levels. We also evaluated the affect of alcohol on TLR8 (CL075) and TLR4 (LPS) receptors and found no significant differences in alcohol-exposed cells (1 or 7 days) both at mRNA (data not shown) or protein levels (Additional file 3, Table S2). This was consistent with our previous finding where we showed that alcohol has no effect on TLR4 expression [28]. This data indicates that alcohol doesn't interfere with receptor expression but might exert its effects on downstream signaling pathways.

### Induction of TNF alpha via TLR8 or TLR4 stimulation is inhibited by acute but enhanced by prolonged alcohol treatment

**Figure 3:**
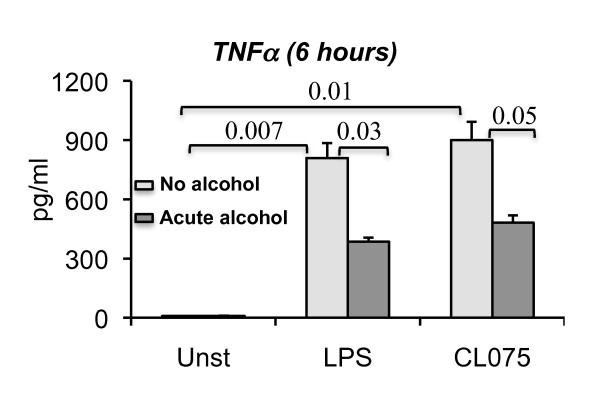
**Acute alcohol treatment down regulates TLR8- or TLR4- induced TNF alpha production**. Human monocytes (n = 3) were either treated or not with 25 mM ethanol or 2.5 ug/ml CL075 (TLR8 ligand) or 0.1 ug/ml LPS (TLR4 ligand) for 6 hours. Some cells were stimulated with a combination of 25 mM ethanol and 2.5 ug/ml CL075 or 25 mM ethanol and 0.1 ug/ml LPS. The cell-free supernatants were collected and TNF alpha production was measured by ELISA. Error bars represent ± SEM. unst: unstimulated.

Blood monocytes contribute to systemic immune responses and represent the source of inflammatory cells recruited to different tissue sites. Here we tested the hypothesis that acute and chronic alcohol regulates the monocyte cytokine production profile in response to TLR8 or TLR4 stimulation. Both LPS, a TLR4 ligand, and CL075, a TLR8 ligand, induced production of the pro-inflammatory cytokine TNF alpha in monocytes isolated from healthy volunteers (Figure 3). We found that co-administration of alcohol as an acute alcohol challenge with TLR8 or TLR4 ligand resulted in a significant inhibition of TNF alpha production (Figure 3). This was consistent with previous studies from our and other laboratories on inhibition of LPS-induced TNF alpha induction by acute alcohol both in vitro and in vivo [28,30-32].

**Figure 4:**
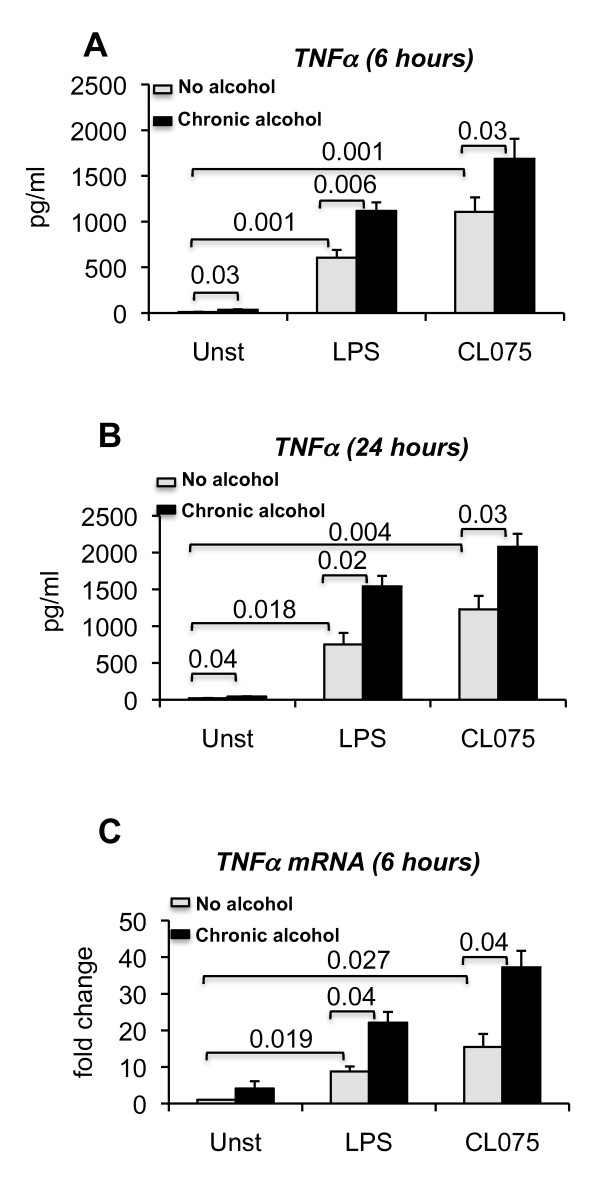
**Prolonged alcohol exposure augments TLR8 or TLR4 induced TNF alpha expression**. Human monocytes (n = 6 or 7) were either treated or not with 25 mM ethanol for 7 days, 2.5 ug/ml CL075 or 0.1 ug/ml LPS for 6 or 24 hours and alcohol treated cells were further stimulated or not with CL075 or LPS for 6 hours (A and C) or 24 hours (B). The cell-free supernatants were collected and analyzed for TNF alpha production by ELISA (A and B, n = 6). RNA was extracted from the cells and real time PCR was performed to quantify TNF alpha gene expression (C, n = 5). Error bars represent ± SEM. unst: unstimulated.

Next, monocytes were exposed to alcohol for 7 days at a physiologically relevant concentration (25 mM), which had no adverse effects on cell viability (Additional file 2, Figure S1). Chronic alcohol treatment alone induced minimal but statistically significant TNF alpha production and robust TNF alpha production was elicited at 6 or 24 hours by TLR8 or TLR4 stimulation (Figure 4A and 4B). In contrast to the effects of acute alcohol, prolonged alcohol treatment significantly augmented both TLR8 and TLR4-induced production of TNF alpha in human monocytes (Figure 4A and 4B). The increase in TNF alpha protein was mirrored by the mRNA upregulation in monocytes after 7 days of alcohol treatment in response to TLR8 or TLR4 stimulation compared to alcohol naïve cells (Figure 4C).

### The anti-inflammatory cytokine, IL-10, is increased by acute alcohol in response to TLR8 or TLR4 stimulation

**Figure 5:**
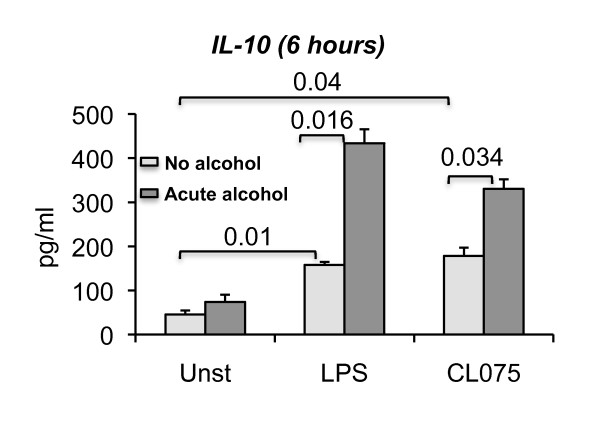
**Acute alcohol exposure augments TLR8- or TLR4- induced IL-10 expression**. Human monocytes (n = 3) were either treated or not with 25 mM ethanol or 2.5 ug/ml CL075 (TLR8 ligand) or 0.1 ug/ml LPS (TLR4 ligand) for 6 hours. Some cells were stimulated with a combination of 25 mM ethanol and 2.5 ug/ml CL075 or 25 mM ethanol and 0.1 ug/ml LPS. The cell-free culture supernatants were collected and IL-10 production was measured by ELISA. Error bars represent ± SEM. unst: unstimulated.

**Figure 6:**
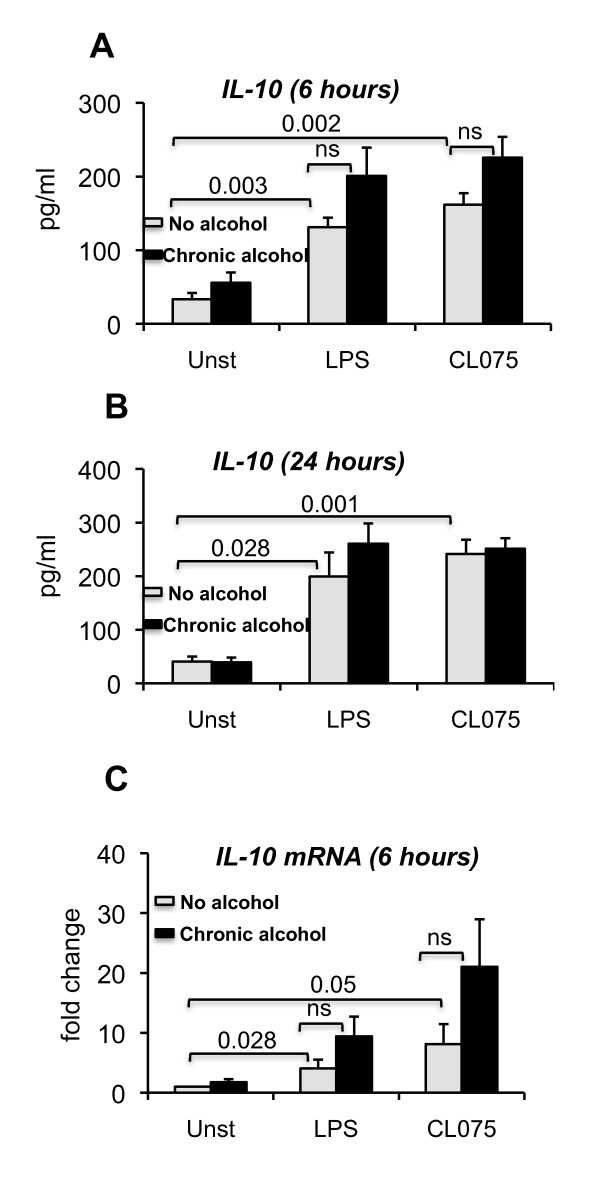
**No significant changes in IL-10 expression after prolonged alcohol treatment**. Human monocytes (n = 6 or 7) were either treated or not with 25 mM ethanol for 7 days, 2.5 ug/ml CL075 or 0.1 ug/ml LPS for 6 or 24 hours and alcohol treated cells were further stimulated or not with CL075 or LPS for 6 hours (A and C) or 24 hours (B). The cell free supernatants were collected and analyzed for IL-10 alpha production by ELISA (A and B, n = 6 or 7). RNA was extracted from the cells and real time PCR was performed to quantify IL-10 gene expression (C, n = 5). Error bars represent ± SEM. unst: unstimulated. ns: non significant.

Pro-inflammatory cytokine production, such as TNF alpha, is counterbalanced by IL-10 induction, an anti-inflammatory cytokine in monocytes [33]. Here we found that acute alcohol treatment significantly upregulated TLR8 and TLR4-induced IL-10 production in human monocytes (Figure 5). The increased IL-10 production in acute alcohol exposed monocytes was consistent with the attenuation of TLR8 and TLR4-induced TNF alpha production (Figure 3). This result suggests the role of IL-10 in the attenuation of TLR8- or TLR4- induced TNF alpha after acute alcohol treatment. Finally, we assessed IL-10 production after prolonged alcohol treatment. While there was a slight tendency for increases in IL-10, there was no significant change in TLR8 or TLR4-induced IL-10 production at the protein (Figure 6A and 6B) or mRNA levels (Figure 6C) in alcohol treated cells.

## Discussion

The immunomodulatory effects of alcohol use have been described in various clinical settings including bacterial and viral infections as well as in post-trauma and post-surgical recoveries [2-4,34]. Increased activation of the inflammatory cascade was found in chronic alcoholics with liver disease where blood monocytes produced increased levels of the pro-inflammatory cytokine TNF alpha [35,36]. While inflammatory cytokine activation has been investigated in several conditions related to alcohol consumption, little is known about the effects of alcohol on the induction of anti-viral immune mediators.

In this study, we investigated the effects of acute and prolonged alcohol on human monocyte responses to TLR8 and TLR4 ligand stimulation and evaluated anti-viral (Type I IFNs), pro- (TNF alpha) and anti-inflammatory (IL-10) cytokine production. Our novel data indicate that IFNβ production in response to TLR8 or TLR4 stimulation was significantly attenuated in monocytes exposed to alcohol. These data suggest that alcohol, regardless of the length of exposure, has inhibitory effects on Type I IFN production in monocytes. Our data further demonstrate that the effects of prolonged alcohol are opposite on Type I IFN induction and inflammatory cytokine induction within the same cell type. Prolonged alcohol exposure inhibited IFNβ production while it increased TLR8- and TLR4-induced TNF alpha production. Finally, we show that the previously described anti-inflammatory effect of acute alcohol on TNF alpha production extends to TLR8-induced TNF alpha induction and involves simultaneous induction of the anti-inflammatory cytokine, IL-10, in human monocytes.

Toll-like receptors are evolutionarily preserved receptors for recognition of danger signals [37]. Nucleic acid sequences in viruses are recognized by TLRs expressed in the endosome including TLR7 and TLR8 that sense single-stranded RNA and TLR9 that sense bacterial DNA [27]. LPS, a component of gram-negative bacteria, activates TLR4 expressed on the cell surface [27]. Human monocytes express a broad repertoire of functionally active TLRs including TLR8 and TLR4 [9,27]. We found that TLR8 or TLR4 ligand stimulation resulted in the induction of both IFNβ and inflammatory cytokines in human blood monocytes. Ligand activation of TLR8 recruits MyD88, a common TLR adapter, and triggers activation of IRAK1/4 kinases and the TRAF6/TAK1 complex leading to the activation of the IKK kinase complex that in turn induces inflammatory cytokines, TNF alpha or IL-10 or phosphorylation of IRFs that induce production of Type I IFNs [38]. TLR4 signaling involves recruitment of the adaptor molecules MyD88 and/or TRIF, each activating different downstream pathways to induce inflammatory cytokines via NF-κB or Type I IFNs via IRF activation, respectively [38]. Considering the differences between the TLR8- and TLR4-induced signaling, our data suggest that alcohol likely modulates multiple components of these signal transduction pathways. Indeed, modulation of NF-κB by acute and chronic alcohol has been extensively studied in the past in LPS-stimulated monocytes [28,39].

The inhibitory effect of alcohol on Type I IFN induction is likely to be a clinically significant finding considering the large number of studies that demonstrate a clinical correlation between alcohol use and impaired anti-viral immune mechanisms [3,17,18,40,41]. Excessive alcohol consumption is known to predispose individuals to secondary infections such as HCV, HIV and bacterial infections [5-7,20]. We found that the inhibitory effects of alcohol occurred both at the IFNβ protein and mRNA levels. As we found no significant changes in TLR8 and TLR4 mRNA or protein after alcohol treatment, we speculate that alcohol interferes with some of the intracellular signaling elements required for IFNβ gene induction in monocytes. Recently, Zhao et al have described the role of PPAR-gamma in limiting IFNβ production via targeting IRF3 in macrophages [42]. In line with this report, alcohol has been shown to activate PPAR-gamma in rats [43] and mice [44].

Previous studies from our and other laboratories showed that IFN-induced signaling pathways are also inhibited by alcohol in blood monocytes as well as in liver cells [19,39,45-47]. Phosphorylation of STAT-1 and STAT-3 was inhibited by acute alcohol suggesting not only IFNβ production but even Type I IFN-induced downstream events could be impaired by alcohol exposure [39]. Recently, we showed that chronic alcohol upregulates microRNA (miRNA)-155 in macrophages [48] therefore, it is reasonable to argue that alcohol might be targeting other miRNAs to limit IFNβ production. In fact, miR-26a, -34a, -145, and let-7b are shown to directly regulate IFNβ in human and macaque cells [49].

We found that acute and prolonged alcohol had opposite effects on both TLR8- and TLR4-induced TNF alpha production in human monocytes. Our laboratory has extensively studied the opposite effects of acute and prolonged alcohol on TLR4-mediated signaling pathways and identified IRAK-M, IRAK1, IKK phosphorylation and NF-κB activation as common targets of acute and chronic alcohol with opposite effects.

We showed that the inhibitory effects of acute alcohol are mediated by alcohol- induced inhibition of NF-κB activation due to decreased phosphorylation of IKKα and IKKβ in LPS-stimulated human monocytes [50]. Based on this information and the fact that TNF alpha induction involves NF-κB activation whether induced via TLR8 or TLR4, our observation of decreased TNF alpha in acute alcohol-exposed TLR8 stimulated monocytes suggests that NF-κB inhibition is a likely mechanism for inhibition of TLR8- induced TNF alpha induction by acute alcohol. An additional mechanism that likely contributes to decreased TNF alpha production after acute alcohol is the increased induction of IL-10 observed in TLR8 or TLR4 stimulated alcohol-exposed monocytes in our experiments. The increased TNF alpha induction by TLR8 ligand in chronic alcohol-exposed monocytes might involve NF-κB activation and reduced IRAK-M by chronic alcohol is also permissive to increase TNF alpha production [28]. Finally, induction of miR-155 after chronic alcohol treatment results in increased TNF alpha production via increasing TNF alpha mRNA stability [48], which is likely the mechanism not only for TLR4- but also TLR8-induced TNF alpha production in monocytes.

Lastly, alcohol treatment (acute or chronic) modulates cytokines that negatively regulate TNF alpha. Previous studies from our laboratory demonstrated that acute alcohol increases IL-10, TGFβ and IL-13 production in human monocytes [51-53]. In a mouse model, acute alcohol has been shown to inhibit TLR- induced inflammatory responses via the p38 and ERK1/2 pathway [54]. While our or others studies found no obvious decrease in the level of anti-inflammatory cytokines after prolonged alcohol exposure in monocytes or in vivo, there is an obvious imbalance between pro- and anti-inflammatory cytokines with the presence of increased TNF alpha and no increase in IL-10 in human monocytes after prolonged alcohol treatment. Thus, it is possible that prolonged alcohol exposure targets multiple signaling molecules to augment TNF alpha expression.

## Conclusions

In summary, to our best knowledge, our data show for the first time that alcohol modulates TLR8-induced monocyte functions both at the level of anti-viral and inflammatory mediator production. Our observations suggest that alcohol exposure, whether acute or prolonged, impairs Type I IFN responses to viral and bacterial PAMPs. The reduced Type I IFN production occurs in the face of increased pro-inflammatory (TNF alpha) cytokine production in monocytes with chronic alcohol exposure suggesting that prolonged alcohol changes the immune balance of monocyte activation.

## Methods

### Human monocyte isolation and reagents

Blood was collected from healthy individuals and monocytes were isolated by the adherence method as described previously [28]. The selection criteria for the individuals signed up for this study was the same as mentioned in our previous study [28] and is approved by the "Institutional Review Board" (IRB), which is called the "Committee for the Protection of Human Subjects in Research" at UMMS. LPS and CL075 were purchased from Sigma-Aldrich and Invivogen respectively. Of note, CL075 mainly induces TLR8, however at high concentrations it can also induce TLR7. The possibility of TLR7 induction in our study is very minimal as monocytes mainly express TLR8 and the concentration of CL075 that we have used in our experiments is very low. Elisa kits for IFNβ (cat # GL80905), TNF alpha (cat # 555212) and IL-10 (Cat # 88-7106) were purchased from Fujirebio Inc, BD Biosciences and eBioscience respectively.

### Cells stimulation

The cells were cultured in 10% IMDM with MEM supplements at 1 × 10^6 ^cell/ml. The serum was obtained from HyClone and its endotoxin level was less than 0.06 EU/ml. Cells were stimulated with 25 mM ethanol for 6 hours (acute) or for 7 days (prolonged) and later challenged or not with 2.5 ug/ml CL075 or 0.1 ug/ml LPS for 6 hours or 24 hours as indicated in the figure legends. For prolonged alcohol treatment (7 days), the cells were incubated in a C.B.S. Scientific chamber with double the alcohol concentration in the bottom of the chamber (to saturate the chamber) and on day 3 or 5, half of the medium was replaced with fresh medium containing alcohol as described previously [28].

Alcohol concentration in the cell-free supernatants was assessed using the Analox Alcohol Analyzer (Analox Instruments).

### Cell viability

Cell viability was checked with an MTT assay (Promega) as per manufacturer's recommendations. Briefly, cells at 1 × 10^6 ^cells/ml were grown in a 96 well plate and treated or not with 25 mM alcohol for indicated times. 15 μl of MTT dye solution was added and incubated at 37°C for ~2 hours in a humidified 5% CO_2 _incubator. After incubation, 100μl of solubilization solution was added and the optical density was measured at 550nm with a reference wavelength of 650nm in a microplate reader (Labsystems). The percentage cell viability was calculated as mean OD of treated cells/mean OD of non- treated cells *100.

### RNA analysis

RNA was isolated with the RNeasy mini kit (Qiagen) and cDNA synthesis was carried out using the QuantiTect transcription kit (Qiagen). The quality of isolated RNA was assessed by spectrophotometric method. Real time PCR was performed (Bio Rad's iCycler iQ real-time detection system) to quantify the expression levels of IFNβ, TNF alpha and IL-10 using gene specific primers. The Ct values were normalized to 18 s (internal control) and fold change was calculated with reference to unstimulated cells. The primers were synthesized from IDT Inc (USA) and the specificity of each primer set was determined by melt curve analysis. The primer sequences were as follows: 18s, forward 5'-GTAACCCGTTGAACCCCATT-3', reverse 5'-CCATCCAATCGG TAGTAGCG-3'; TNFα, forward 5'-ATCTTCTCGAACCCCGAGTGA-3', reverse 5'- CGGTTCAGCCACTGGAGCT-3', IL-10, forward 5'- AGACCCAGACATCAAGGC GCA-3', reverse 5'-ATCGATGACAGCGCCGTAGCC-3', IFNβ, forward 5'-TCATA TGCAGTACATTAGCCATCA -3', reverse 5'-GATCTCCTAGCCTGTGCCTC-3'.

### ELISA

The amount of IFNβ, TNF alpha and IL-10 in cell-free supernatants was measured by ELISA as per manufacturer's instructions.

### Flow cytometry analysis

Isolated monocytes were rested for one day and next day either stimulated or not with 25 mM alcohol for indicated times. At the end of stimulation, cells were used for staining using standard staining methods. Briefly, cells were collected, washed and incubated with fluorochrome-conjugated TLR4 (Biolegend, cat# 312805) and CD14 or isotype control Abs at 4°C for 30min, washed twice in PBS containing 2% FBS and fixed in 1% paraformaldehyde. For TLR8 staining, cells were first stained for cell surface CD14 protein and then permeabilized using a cell permeabilization kit (BD biosciences), and incubated with TLR8 Ab (Imgenex, cat# IMG-321C) and later fixed. Data was collected using a FACScan II flow cytometer (BD Biosciences), and analyzed using FlowJo software (Tree Star, Inc.). The fluorochrome-conjugated Abs against human cell surface protein CD14 (cat# 555399) and isotype controls were purchased from BD Pharmingen.

### Statistical analysis

Statistical analysis was carried out by student t test (two tailed) and p value less than 0.05 was considered significant.

## Authors' contributions

GS and SB conceived the idea and outlined the project. MP, DC and KK carried out the experiments. GS, SB, DC and KK contributed to data analysis, writing and editing of the manuscript. All the authors read and approved the final manuscript.

## Supplementary Material

Additional file 1 **Table S1**. Alcohol concentration in the supernatants of cultured human monocytes. Click here for file

Additional file 2 **Figure S1. Alcohol treatment does not affect cell viability**. Human monocytes (n = 2) were either treated or not with 25 mM alcohol for indicated times in a 96 well plate at 1 × 10^6 ^cell/ml. Cell viability was assessed by an MTT kit as per manufacture's instruction and optical density was measured at 550 nm with a reference wavelength of 650 nm. The percentage cell viability was calculated as mean OD of treated cells/mean OD of non-treated cells *100. Error bars represent mean ± SEM. unst: unstimulated, ns: non significant. Click here for file

Additional file 3 **Table S2**. Flow cytometric analysis of TLR4 and TLR8 proteins. Click here for file
