# Supplementary material for: Inhibition of TLR8- and TLR4-induced Type I IFN induction by alcohol is different from its effects on inflammatory cytokine production in monocytes
Source: BMC Immunol. 2011 Sep 30;12:55. doi: 10.1186/1471-2172-12-55 (PMC3203086; doi:10.1186/1471-2172-12-55)
Supplement: Additional file 1 — Table S1. Alcohol concentration in the supernatants of cultured human monocytes. [file 1471-2172-12-55-S1.PDF]

Table S1. Alcohol concentration in the supernatants of cultured Human monocytes

	Ethanol level (mM)
Day1	23.0
Day2	20.7
Day3	23.0
Day4	21.3
Day5	22.5
Day6	21.4
Day7	20.9
