# Supplementary figures and images for: Inhibition of TLR8- and TLR4-induced Type I IFN induction by alcohol is different from its effects on inflammatory cytokine production in monocytes
Source: BMC Immunol. 2011 Sep 30;12:55. doi: 10.1186/1471-2172-12-55 (PMC3203086; doi:10.1186/1471-2172-12-55)

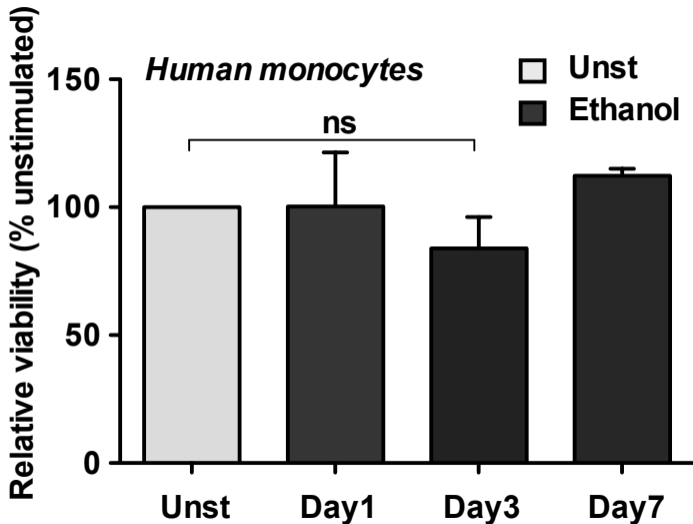

Supplement: Additional file 2 — Figure S1. Alcohol treatment does not affect cell viability. Human monocytes (n = 2) were either treated or not with 25 mM alcohol for indicated times in a 96 well plate at 1 × 10^6^ cell/ml. Cell viability was assessed by an MTT kit as per manufacture's instruction and optical density was measured at 550 nm with a reference wavelength of 650 nm. The percentage cell viability was calculated as mean OD of treated cells/mean OD of non-treated cells *100. Error bars represent mean ± SEM. unst: unstimulated, ns: non significant. [file 1471-2172-12-55-S2.PDF]
