# Supplementary material for: Inhibition of TLR8- and TLR4-induced Type I IFN induction by alcohol is different from its effects on inflammatory cytokine production in monocytes
Source: BMC Immunol. 2011 Sep 30;12:55. doi: 10.1186/1471-2172-12-55 (PMC3203086; doi:10.1186/1471-2172-12-55)
Supplement: Additional file 3 — Table S2. Flow cytometric analysis of TLR4 and TLR8 proteins. [file 1471-2172-12-55-S3.PDF]

Table S2. Flow cytometric analysis of TLR4 and TLR8 proteins

**Markers**	**Average of mean fluorescent intensity (MFI), n=3**			**p**
	Unstimulated	25mM ethanol Day1	25mM ethanol Day7	
TLR4	10.3±1.38	9.8±2.2	18.0±7.93	ns
TLR8	369±163.4	416±80	627±290	ns
